# Endotoxin Engages Mitochondrial Quality Control *via* an iNOS-Reactive Oxygen Species Signaling Pathway in Hepatocytes

**DOI:** 10.1155/2019/4745067

**Published:** 2019-10-24

**Authors:** Anthony Cyr, Lauran Chambers, Paul K. Waltz, Sean P. Whelan, Lauryn Kohut, Evie Carchman, Mitchell Dyer, Jason Luciano, Benjamin Kautza, Hernando D. Gomez, Leo E. Otterbein, Matthew R. Rosengart, Sruti Shiva, Brian S. Zuckerbraun

**Affiliations:** ^1^Department of Surgery, University of Pittsburgh, Pittsburgh, Pennsylvania, USA; ^2^Department of Surgery, University of Wisconsin-Madison, Madison, Wisconsin, USA; ^3^Department of Critical Care Medicine, University of Pittsburgh, Pittsburgh, PA, USA; ^4^Department of Surgery, Beth Israel-Deaconess Medical Center, Boston, MA, USA; ^5^Department of Pharmacology, University of Pittsburgh, Pittsburgh, PA, USA; ^6^Vascular Medicine Institute, University of Pittsburgh, Pittsburgh, PA, USA; ^7^VA Pittsburgh Healthcare System, USA

## Abstract

**Background:**

Organ injury and dysfunction in sepsis accounts for significant morbidity and mortality. Adaptive cellular responses in the setting of sepsis prevent injury and allow for organ recovery. We and others have shown that part of the adaptive response includes regulation of cellular respiration and maintenance of a healthy mitochondrial population. Herein, we hypothesized that endotoxin-induced changes in hepatocyte mitochondrial respiration and homeostasis are regulated by an inducible nitric oxide synthase/nitric oxide (iNOS/NO)-mitochondrial reactive oxygen species (mtROS) signaling axis, involving activation of the NRF2 signaling pathway.

**Methods:**

Wild-type (C57Bl/6) or iNos^−/−^ male mice were subjected to intraperitoneal lipopolysaccharide (LPS) injections to simulate endotoxemia. Individual mice were randomized to treatment with NO-releasing agent DPTA-NONOate, mtROS scavenger MitoTEMPO, or vehicle controls. Other mice were treated with scramble or *Nrf2*-specific siRNA *via* tail vein injection. Primary murine hepatocytes were utilized for *in vitro* studies with or without LPS stimulation. Oxygen consumption rates were measured to establish mitochondrial respiratory parameters. Western blotting, confocal microscopy with immunocytochemistry, and rtPCR were performed for analysis of iNOS as well as markers of both autophagy and mitochondrial biogenesis.

**Results:**

LPS treatment inhibited aerobic respiration *in vitro* in wild-type but not *iNos*^−/−^ cells. Experimental endotoxemia *in vivo* or *in vitro* induced iNOS protein and mtROS production. However, induction of mtROS was dependent on iNOS expression. Furthermore, LPS-induced hepatic autophagy/mitophagy and mitochondrial biogenesis were significantly attenuated in *iNos*^−/−^ mice or cells with NO or mtROS scavenging. These responses were rescued in *iNos*^−/−^ mice *via* delivery of NO both *in vivo* and *in vitro. Conclusions*. These data suggest that regulation of mitochondrial quality control following hepatocyte LPS exposure is dependent at least in part on a NO-mtROS signaling network. Further investigation to identify specific agents that modulate this process may facilitate the prevention of organ injury in sepsis.

## 1. Introduction

Severe sepsis represents a major healthcare burden in the United States with annual case incidence exceeding 750,000 and an associated mortality of nearly 30% [1]. Sepsis results in a complex host response involving both inflammatory and anti-inflammatory pathway activation, driven by the interaction of pathogen-associated molecular patterns (PAMPs) and the host's pattern recognition receptors (PRRs) [2]. Despite multiple advances in both basic and clinical research on sepsis, therapy remains largely supportive focusing on judicious fluid resuscitation, control of the underlying source, and appropriate antimicrobial therapy [3].

While the underlying pathophysiology in sepsis is undoubtedly multifactorial and heterogeneous, one commonly accepted feature is the notion of “cytopathic dysoxia.” This refers to an energy deficit experienced at the cellular level stemming from impaired utilization of oxygen by the mitochondria, and not necessarily from a deficit in oxygen delivery to the cell [4]. For example, evaluation of renal histology in the setting of sepsis demonstrates minimal and focal cell death incommensurate with the amount of organ dysfunction present; however, at the organelle level, there is evidence of hydropic mitochondria on electron microscopy [5]. More broadly, in a rat model of peritonitis, the severity of organ dysfunction was found to correlate with the degree of mitochondrial dysfunction and the overproduction of nitric oxide (NO) [6]. These findings implicate the mitochondria as important mediators of the clinical consequences of sepsis [7].

Multiple cellular adaptive responses exist to minimize damage and restore homeostasis. Autophagy is a mechanism in which damaged or unnecessary intracellular contents are targeted and degraded by lysosomes. Autophagy of mitochondria, deemed mitophagy, may exist as a means to clear damaged mitochondria and minimize subsequent cellular stress [8]. Autophagy has been shown to be upregulated in hepatocytes in both experimental and clinical sepsis [9, 10]. LC3 levels (a key marker in autophagy), in particular, have been shown to be upregulated in surviving patients of critical illness (sepsis and multiorgan failure) versus those who do not survive [11, 12]. On the other hand, mitochondrial biogenesis is a conserved process that contributes to the restoration of a healthy pool of mitochondria [8], and biogenesis is seemingly critical to preventing organ damage [7, 9]. Compared to those who do not survive critical illness, survivors experienced 2.5-fold increases in the mitochondrial biogenesis-associated genes peroxisome proliferator-activated receptor gamma coactivator 1-*α* (PGC-1*α*) and nuclear respiratory factor 1 (NRF1) expression when compared to nonsurvivors, supporting biogenesis as a means of organ damage prevention [13]. In addition, stress-induced signaling pathways that influence mitochondrial function are likely to play a role in sepsis-induced changes in respiratory rates and maintenance of the mitochondrial network. In particular, iNOS induction and subsequent NO production as well as heme oxygenase-1 (HO-1) induction and associated carbon monoxide (CO) production influence mitochondrial respiratory function, mitophagy, and biogenesis in sepsis [11, 14, 15]. Taken together, it is clear that mitochondrial quality control could play a major role in determining individual patient outcomes following critical illness. In this context, the purpose of these investigations was to test the hypothesis that endotoxemia modulates hepatic bioenergetics via regulation of mitochondrial respiration by alteration of mitochondrial quality control pathways and is dependent on an iNOS/NO-mtROS signaling network.

## 2. Materials and Methods

### 2.1. Cell Culture

Primary hepatocytes were harvested from C57BL/6 (Jackson Laboratories, Bar Harbor, ME, USA) or *iNOS*^−/−^ mice as described previously [16, 17]. Cells were utilized on days 1-3 after harvest. LPS treatment in all *in vitro* hepatocyte experiments was at a concentration of 100 ng/mL for at times specified in the text. Cells were maintained at 37°C, 21% O_2_, and 5% CO_2_. HepG2 cells were purchased from ATCC and cultured in Dulbecco's modified Eagle's medium (DMEM) supplemented with 10% fetal bovine serum and 1% penicillin/streptomycin. HepG2 *ρ*0 cells were generated using long-term, low-dose ethidium bromide treatment and the gradual loss of mitochondrial DNA was monitored through serial PCR, as previously demonstrated. For studies of antioxidant therapy, cells were cultured in 5 *μ*M MitoTEMPO for times indicated in text. Cells were additionally cultured in the presence of highly selective iNOS inhibitor 1400 W at 10 nM for times indicated in the text or figure legends.

### 2.2. Animal Studies

Animal protocols were approved by the University of Pittsburgh Institutional Animal Care and Use Committee. The experiments were performed in adherence to the US National Institutes of Health guidelines on the use of laboratory animals. Endotoxemia was induced via intraperitoneal injection at (5 mg/kg) on C57BL/6 or *iNos*^−/−^ mice ages 8-12 weeks, weighing 20-25 g. Blood and tissue were harvested 12 hours following LPS administration. No antibiotics were given. Animals had free access to food and water before and after the procedure. For *in vivo* experiments with the NO donor DPTA-NONOate, animals were given 10 mg/kg intraperitoneally at the time of LPS exposure. Other animals were dosed with the mtROS scavenger MitoTEMPO at 1 mg/kg *via* intraperitoneal injection, given 4 hours prior to sacrifice.

### 2.3. siRNA Treatment

*Nrf2* expression was knocked down *in vivo* with specific siRNA (Invitrogen; 50 *μ*g/kg). This was administered by hydrodynamic tail vein injection where the appropriate dose of siRNA was delivered in a total of 2 mL of lactated Ringer's solution, given three days prior to LPS administration. This rapid injection of high volume produces significant pressure to promote siRNA uptake intracellularly. Scramble siRNA (50 *μ*g/kg) was used as a control. *Nrf2* expression was knocked down *in vitro* using manufacturer's specifications for transfection with Lipofectamine (Invitrogen). Cells were subsequently treated with LPS or control 24 h following siRNA transfection.

### 2.4. Immunocytochemistry/Immunohistochemistry

Cells were fixed on coverslips with 2% paraformaldehyde for 15 minutes and then rinsed with cold PBS. Liver tissue harvested from mice was removed after whole body perfusion with cold PBS followed by 2% paraformaldehyde. Tissue was placed in 2% paraformaldehyde for 1 hour and then switched to 30% sucrose in distilled water for 12 hours. Tissue was then slowly frozen in 2-methylbutane. Tissue sections were obtained at 7 *μ*m and were then stained using 2′-(4-hydroxyphenyl)-5-(4-methyl-1-piperazinyl)-2,5′-bi(1H-benzimidazole) trihydrochloride (Sigma, cat no. B-2883) for nuclear identification and 488-conjugated phalloidin antibody (BioLegend, cat no. 424201) for actin cytoskeletal identification. Specific antibodies to LC3 (Novus, St. Charles, MO, USA), PGC-1a (Abcam, Cambridge, MA, USA), NRF1, NRF2, or HO-1 (Abcam, Cambridge, MA, USA) were additionally utilized for imaging as indicated according to manufacturer's instructions. All slides were scanned under the same conditions for magnification, exposure time, lamp intensity, and camera gain. Confocal images were acquired using the Nikon A1 with a PlanApo N (×20 with and without a 2-fold digital zoom). All imaging studies were repeated at least *n* = 3 times on biological replicates.

### 2.5. Measurement of Reactive Oxygen Species

For *in vivo* evaluation of mitochondrial reactive oxygen species production, mice received MitoSOX (Thermo Fisher, Rockford, IL, USA, 2 mg/kg) as an intraperitoneal injection 60 minutes prior to euthanasia. Tissues were then subsequently harvested as described above and analyzed for MitoSOX fluorescence, which indicates oxidation. For *in vitro* experiments, cells were subjected either to MitoSOX treatment in culture or cell-permeant 2′,7′-dichlorodihydrofluorescein diacetate (H_2_DCFDA). For the latter, the fluorescence of the oxidized analog 2′,7′-dichlorofluorescein (DCF) was measured as an analog of whole cell ROS production.

### 2.6. Western Blot

Hepatocytes were washed with cold PBS, collected in cell lysis buffer, sonicated, and centrifuged (10,000g for 15 min), and the protein-rich supernatant was transferred to a new tube. Protein concentrations were calculated using the BCA protein assay kit (Pierce Biotechnology, Rockford, IL). Samples were then mixed with loading buffer and run on an SDS-polyacrylamide gel. This gel was then transferred to a cellulose membrane. The membrane was blocked in 5% milk in TBS-Tween 20 for 1 hour and then incubated in primary antibodies. Antibodies utilized were iNOS, NRF2 (Abcam, Cambridge, MA), LC3 (Novus Biologicals, Littleton, CO, USA), and *β*-actin (Abcam). Membranes were then washed in TBS-Tween 20 for 30 minutes, placed in secondary antibody for 1 hour, and then washed for 1 hour in TBS-Tween 20 prior to being developed using chemiluminescence substance (Thermo Fisher, Rockford, IL, USA).

### 2.7. rtPCR

Cells were cultured as described. RNA was prepared using the RNeasy Midi Kit (Qiagen, Valencia, CA, USA), according to the manufacturer's instructions. An on-column DNase digestion using RNase-free DNase (Qiagen) was performed to eliminate genomic DNA contamination. RNA (1 *μ*g) was used to generate cDNA using oligo dT primers and Omniscript (Qiagen) reverse transcriptase. PCR reaction mixtures were prepared using SYBR green PCR master mix (PE Applied Biosystems, Foster City, CA, USA). SYBR green 2-step real-time RT-PCR for *Pgc-1α*, *Nrf1*, and *Tfam* was performed. All samples were run in duplicate. The level of gene expression for each sample was normalized to the *β*-actin mRNA expression using the comparative C_t_ method.

### 2.8. Mitochondrial Complex Activity

Complex II activity was determined by measuring the reduction of dichloroindophenol (DCIP) at 600 nm, which was coupled to the oxidation of CoQ_2_ using succinate as a substrate. Thenoyltrifluoroacetone (TTFA) was used to determine specificity of the assay for complex II activity.

### 2.9. Oxygen Consumption

Hepatocytes from wild-type or *iNOS*^−/−^ mice were plated at a density 20,000 cells/well on XF24 cell culture plates (Seahorse Biosciences, North Billerica, MA, USA) in a final volume of 250 *μ*L. Hepatocytes were then treated with LPS (100 ng/mL) for varying lengths of time. These cells were then rinsed with unbuffered DMEM, placed in 37°C incubator without CO_2_ for 1 hour, and then loaded onto the XF24 instrument. Oxygen consumption rates and extracellular acidification were measured according to standard instrumental protocols. Each condition was run in quadruplicate, and each well was read 8 times. Experiments were repeated 3 times.

### 2.10. Statistical Analysis

Results are expressed as mean ± standard error of the mean (SEM). SigmaPlot (Systat Software, Inc., Point Richmond, CA) was used for the statistical analysis using either Student's *t*-test for pairwise comparisons or one-way analysis of variance (ANOVA) for significance and Tukey's post hoc test. Significance was established as *p* < 0.05. All *in vitro* experiments were performed in triplicate and repeated three times unless specified otherwise. All *in vivo* experiments contained 6-8 mice per group as specified.

## 3. Results

### 3.1. LPS Induces iNOS to Influence Hepatocyte Respiration

**Figure 1:**
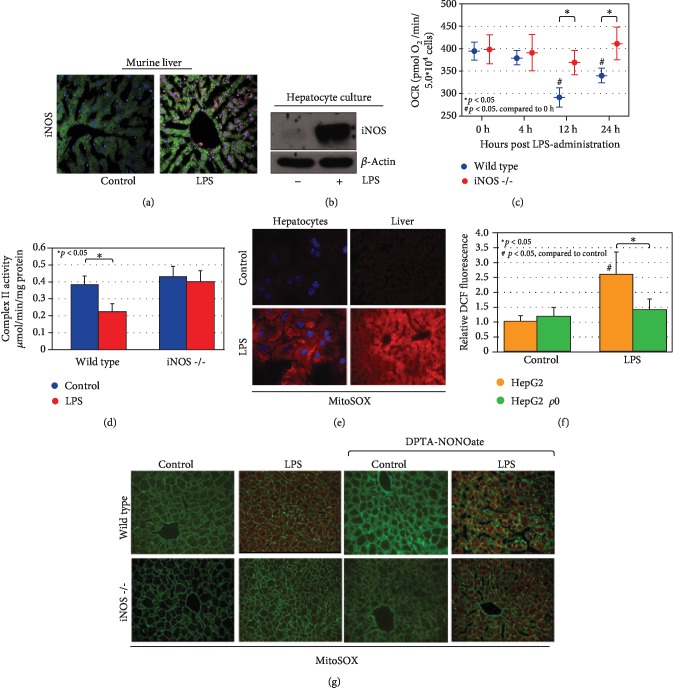
LPS induces mitochondrial dysfunction and mtROS production in an iNOS-dependent fashion. LPS induction leads to iNOS activation both *in vivo* (at 12 h exposure, (a)) and in cultured hepatocytes (following 6 h exposure, (b)). Cultured hepatocytes with prolonged exposure to LPS (times indicated) demonstrate a mitochondrial oxygen consumption defect over time that is dependent on iNOS expression (c). Evaluation of specific complex II activity in hepatocytes demonstrates a similar iNOS-dependent reduction of activity following LPS exposure (single time point, 12 h, (d)). In conjunction, LPS exposure is associated with increased mtROS production as measured by MitoSOX both *in vivo* and *in vitro* (12 h LPS exposure, 60 min preloading of MitoSOX, (e)). Global ROS production as measured by DCF fluorescence is predominantly mitochondrial in origin, as HepG2 *ρ*0 cells (which lack mitochondrial DNA) do not significantly generate ROS following LPS stimulation in comparison with HepG2 parent cells (12 h LPS exposure, (f)). MtROS generation following LPS administration as detected through *in situ* MitoSOX staining in liver sections is dependent on iNOS. MtROS production can be reconstituted in iNos^−/−^ hepatocytes with the NO donor DPTA-NONOate, suggesting NO signaling downstream of iNOS is critical (12 h LPS and/or DPTA-NONOate, 60 min preloading of MitoSOX, (g)). In immunofluorescent imaging, green staining is from cytoskeletal actin as measured by 488-conjugated phalloidin antibody. Statistical significance is highlighted in individual panels as necessary.

LPS administration *in vitro* or *in vivo* resulted in increased hepatic iNOS protein levels (Figures 1(a) and 1(b)). The role of iNOS in the regulation of bioenergetics in liver or hepatocytes in sepsis models was next investigated. Previous studies demonstrated that LPS or experimental sepsis results in a transient depression of oxidative phosphorylation—a similar phenomenon to the Warburg effect in cancer cells [18]. The present data demonstrate that LPS-associated reduction in the rate of oxygen consumption in hepatocytes was dependent on iNOS expression (Figure 1(c)). Oxygen consumption rate (OCR) decreased from 395 ± 20 to 292 ± 21 and 341 ± 16 at 12 and 24 hours, respectively following LPS treatment in wild-type hepatocytes (*p* < 0.05 compared to 0 h OCR). Importantly, LPS treatment did not influence cell number or viability at across the time course and treatment dose chosen (data not shown). Conversely, OCR changed minimally in hepatocytes from *iNos*^−/−^ mice (399 ± 32 to 370 ± 27 and 412 ± 36 at 12 and 24 hours respectively; *p* < 0.05 compared to wild-type hepatocytes at identical time points). Furthermore, LPS-induced reductions in complex II activity were dependent on iNOS expression (Figure 1(d)). LPS decreased complex II activity in hepatic tissue from 0.384 ± 0.05 to 0.223 ± 0.048 *μ*mol/min/mg (*p* < 0.05). No significant changes were noted upon LPS stimulation in hepatocytes in either complex I or complex IV activity (data not shown). Mirroring the OCR, complex II activity was not decreased by LPS in *iNos^−/−^* mice (0.431 ± 0.06 to 0.399 ± 0.07 *μ*mol/min/mg). These data suggest that respiratory depression of mitochondria seen in response to LPS is in part dependent on iNOS expression.

### 3.2. LPS-Induced Hepatic mtROS Is Dependent on iNOS Expression

LPS *in vitro* or *in vivo* induced mtROS production as measured by MitoSOX fluorescence (Figure 1(e)). Additionally, LPS increased the nonspecific ROS marker DCF fluorescence in HepG2 hepatocytes but not in *ρ*0 HepG2 cells, which lack mitochondrial DNA (2.63 ± 0.75-fold versus 1.46 ± 0.33, Figure 1(f)), suggesting that the ROS generation is mitochondrial in origin. The dependence of LPS-induced mtROS on iNOS expression was subsequently determined *in vivo.* LPS failed to increase MitoSOX fluorescence in *iNos*^−/−^ mice. However, ROS production could be restored in *iNos*^−/−^ mice with concurrent treatment with the NO donor DPTA-NONOate (Figure 1(g)).

### 3.3. Mitochondrial Quality Control Pathways Are Induced in an iNOS-Dependent Fashion following LPS Exposure

**Figure 2:**
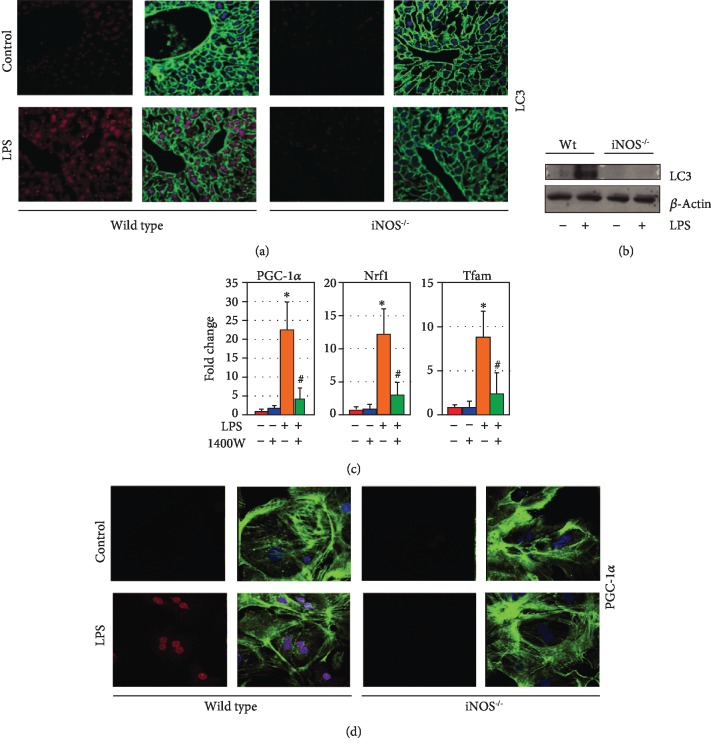
Mitochondrial quality control in hepatocytes following LPS stimulation is dependent on iNOS induction. Autophagy marker LC3 (red) is induced in hepatocytes in an iNOS-dependent fashion following LPS exposure *in vivo* (12 h exposure, (a)) and *in vitro* (12 h exposure, (b)). Inhibition of iNOS in hepatocytes with the chemical inhibitor 1400 W (10 nM, 12 h cotreatment with LPS) suppresses induction of mitochondrial biogenesis-associated genes *Pgc-1α, Nrf1*, and *Tfam* following LPS expression as measured by q-RT-PCR (12 h LPS exposure, (c)). PGC-1*α* is induced in cultured hepatocytes following LPS exposure in an iNOS-dependent fashion (red staining, 12 h LPS exposure, (d)). ^∗^*p* < 0.05 compared to untreated controls; ^#^*p* < 0.05 compared to LPS-treated alone. In immunofluorescent imaging, green staining is from cytoskeletal actin as measured by 488-conjugated phalloidin antibody.

We and others have shown that protection against cell death in the setting of sepsis is in part dependent on autophagy and mitochondrial biogenesis [11]. This prior work has also established a complex interplay between mitophagy and the process of mitochondrial biogenesis to maintain a healthy mitochondrial network, with mitochondrial biogenesis being in part dependent on mitophagy. In the present work, the induction of autophagy and mitochondrial biogenesis by LPS was determined. Consistent with previous findings, LPS resulted in increased hepatocyte LC3 punctae and protein levels as a measure of increased autophagic signaling. However, minimal changes were demonstrated in *iNos*^−/−^ mice compared to wild-type mice (Figures 2(a) and 2(b)). Similarly, LPS increased markers of mitochondrial biogenesis *in vitro* and *in vivo*, and this was diminished in the setting of genetic deletion of iNOS expression or the pharmacological iNOS inhibitor 1400 W. Relative (compared to actin) expression profiles of mitochondrial biogenesis genes *Pgc*-1*α*, *Nrf1*, and mitochondrial transcription factor a (*Tfam*) were increased in LPS-treated hepatocytes compared to non-LPS hepatocytes by 22.5 ± 7.3, 12.4 ± 3.6, and 8.8 ± 3.0, respectively (*p* < 0.05 compared to untreated controls, Figure 2(c)). Inhibition of iNOS by 1400 W limited the increase in RNA expression to 4.1 ± 2.8, 2.9 ± 2.1, and 2.3±2.0, respectively (*p* < 0.05 compared to LPS-only treated, Figure 2(c)). LPS-induced hepatocyte protein expression of PGC-1*α* was also limited in *iNos*^−/−^ cells by immunocytochemistry (Figure 2(d)).

### 3.4. LPS Induces NRF2 to Modulate Mitochondrial Quality Control in an iNOS-Dependent Fashion

**Figure 3:**
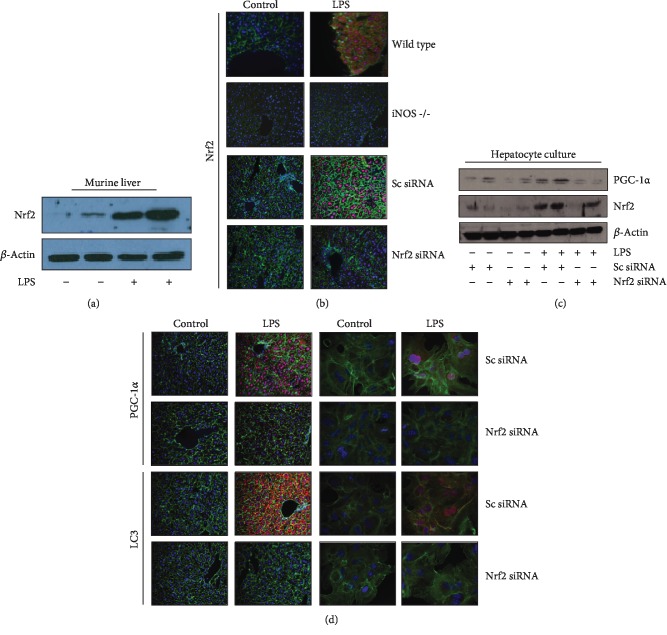
iNOS-dependent Nrf2 induction is required for mitochondrial quality control process activation following LPS stimulation. Nrf2, a critical regulator of mitochondrial quality control, is induced following LPS exposure *in vivo* in murine liver (12 h LPS exposure, (a)). Nrf2 induction following LPS stimulation is dependent on iNOS in murine liver, and Nrf2 expression can successfully be knocked down *in vivo* using siRNA (protocol in methods, (b)). In cultured hepatocytes and *in vivo* murine liver, Nrf2 expression is required for appropriate induction of PGC-1*α* following LPS exposure as measured by Western blot (cultured hepatocytes, 12 h LPS exposure, (c)) and confocal microscopy (red stain, (d) upper section). Appropriate expression of autophagy protein LC3 following 12 h LPS exposure is similarly dependent on Nrf2 both *in vivo* and *in vitro* (red stain, (d) lower section). In immunofluorescent imaging, green staining is from cytoskeletal actin as measured by 488-conjugated phalloidin antibody, whereas blue nuclear staining is from 2′-(4-hydroxyphenyl)-5-(4-methyl-1-piperazinyl)-2,5′-bi(1H-benzimidazole) trihydrochloride.

Nuclear factor erythroid 2-related factor 2 (NRF2) is a well-characterized signaling node, induced by prooxidant stressors, that plays a role in regulating mitochondrial quality control [19]. NRF2 protein was induced by LPS administration *in vivo* (Figure 3(a)). This effect was dependent on iNOS expression, as demonstrated by immunocytochemistry in both wild-type and *iNos*^−/−^ liver sections following LPS exposure (Figure 3(b), upper panels). Furthermore, NRF2 expression could successfully be knocked down by tail-vein injection of anti-*Nrf2* siRNA (Figure 3(b)). Downstream expression of both PGC-1*α* and LC3 was determined to be dependent on NRF2 expression as well, both by Western blotting in primary hepatocyte culture (Figure 3(c)) and through immunocytochemistry both from *in situ* liver sections (Figure 3(d), left panels) and primary hepatocyte culture (Figure 3(d), right panels). These data demonstrate the necessity of NRF2 expression downstream of iNOS signaling following LPS stimulation.

### 3.5. LPS Induction of NRF2 and Mitochondrial Quality Control Is Dependent on iNOS-Mediated mtROS Production

**Figure 4:**
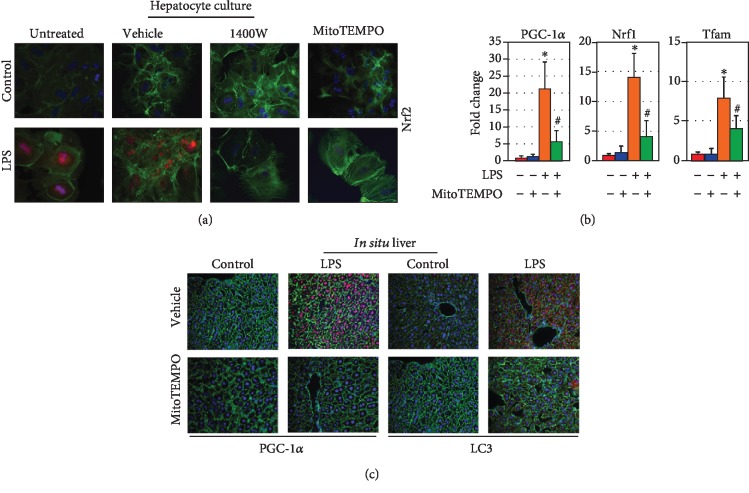
mtROS signaling following LPS stimulation is required for induction of mitochondrial quality control pathways in hepatocytes. In hepatocyte culture, Nrf2 expression following LPS stimulation is abrogated by both the iNOS inhibitor 1400 W (10 nM) and the mitochondrial ROS scavenger MitoTEMPO (5 *μ*M, 12 h LPS exposure, (a)). Induction of the mitochondrial biogenesis regulatory genes *Pgc-1α*, *Nrf1*, and *Tfam* as measured by qRT-PCR following 12 h LPS exposure was suppressed by co-culture with MitoTEMPO (5 *μ*M), suggesting a partial dependence on mtROS (b). mtROS scavenging with MitoTEMPO (1 mg/kg) *in vivo* also abrogates appropriate induction of both PGC-1*α* and LC3 following LPS administration (12-hour exposure, (c)). ^∗^*p* < 0.05 compared to untreated controls; ^#^*p* < 0.05 compared to LPS-treated alone.

We next sought to evaluate the role of mtROS production downstream of iNOS following LPS stimulation in hepatocytes. We confirmed in cultured wild-type hepatocytes that administration of iNOS inhibitor 1400 W could successfully suppress NRF2 expression *via* immunocytochemistry (Figure 4(a)). Additionally, NRF2 expression could be suppressed through treatment with the mtROS scavenger MitoTEMPO (Figure 4(a)). Given this, we sought to evaluate the role of mtROS scavenging in the expression profiles of mitochondrial biogenesis genes. Relative expression of *Pgc-1α*, *Nrf1*, and *Tfam* (normalized to actin) was increased in LPS-treated hepatocytes compared to non-LPS hepatocytes by 21.4 ± 7.9, 14.2 ± 4.1, and 8.1 ± 2.7, respectively (*p* < 0.05; Figure 4(b)). MitoTEMPO pretreatment limited expression increase with LPS to 5.9 ± 3.1, 4.3 ± 2.7, and 4.1 ± 1.8, respectively (*p* < 0.05 compared to LPS-treated alone, Figure 4(b)). Furthermore, MitoTEMPO was able to prevent appropriate induction of PGC-1*α* and LC3 protein following LPS exposure as measured by immunocytochemistry in liver sections from treated mice (Figure 4(c)).

## 4. Discussion

Taken together these data show that iNOS is upregulated on exposure to LPS, stimulating increased mitochondrial biogenesis and autophagy in an mtROS-NRF2-dependent manner. This response may be an important method of regulating bioenergetics in hepatocytes in the setting of clinical sepsis.

Gasotransmitters that regulate mitochondrial homeostasis and respiration are likely important regulators of the bioenergetic response to sepsis. Increased iNOS expression and activity is well described in response to LPS and infection. NO is known to decrease electron flux through the electron transport chain and increase mtROS. These data demonstrate that following LPS treatment of hepatocytes, NO is critical in regulating oxidative phosphorylation and the generation of ROS production to influence mitochondrial adaptive responses. The induction of iNOS in hepatocytes is mediated by the pattern recognition receptor TLR-4. This rudimentary stress response and ability of NO to posttranslationally modify a platitude of proteins, including protein complexes of oxidative phosphorylation, may allow for immediate and broad signaling throughout a cell to prime for the stress of infection. Increased production of NO can be detrimental to cells through oxidative or nitrosative stress. Additionally, models of neurodegeneration have shown increased NO to inhibit autophagy which may potentially lead to increased cell stress [20]. Our results here in iNos^−/−^ mice show that there is dependence on intact iNOS for LPS-induced autophagic and mitochondrial biogenesis signaling in the liver and hepatocytes. We did not directly assess eNOS activity, an additional known regulator of mitochondrial biogenesis [21]. Notably, we also did not directly assess the effect of NO donors or NOS activators directly on mitochondrial biogenesis in the absence of LPS signaling. We did not pursue this specifically in the current set of experiments based on our early findings (Figure 1(g)) that NO donors did not appear to impact MitoSOX fluorescence patterns either in control or *iNos*^−/−^ livers. While MitoSOX is a blunt instrument for detecting mitochondrial ROS, other findings in our paper demonstrated that the abrogation of mitochondrial ROS using MitoTEMPO abrogated the signaling cascade leading to mitochondrial biogenesis processes (Figure 4). This in turns suggests that NO donor therapy alone would be insufficient for activating this cascade given the lack of mitochondrial ROS induction. Broadly, there is likely a complex balance affording protective cellular responses mediated by NO, as well as cell-type-specific responses.

The regulation of mitochondrial biogenesis is complex. Biogenesis is part of multiple dynamic responses aimed at maintaining a healthy mitochondrial population. The ultimate goal is a balanced response of mitochondrial biogenesis and mitophagy to maintain an adequate pool of mitochondria, either too much biogenesis or insufficient mitophagy can result in increased cell stress and diminished function [8]. In our hepatocyte model, iNOS was required for the appropriate induction of the mitochondrial quality control mechanisms, in an mtROS-dependent fashion. This highlights the underlying importance of appropriate balance in signaling cascades that govern responses to sepsis. For example, prior studies demonstrated that both complex I and complex IV assemblies were downregulated in skeletal muscle fibers in patients with critical illness, with associated proportional changes in citrate synthase activity. When assessing survivors vs. nonsurvivors among this cohort, a larger degree of mitochondrial swelling was noted in survivors than nonsurvivors, in addition to a higher degree of mRNA expression of Sod2, Pgc-1*α*, Tfam, and Nrf1 [13]. Although our work makes no claims about survival following endotoxemic insult, it does help clarify some of the underlying mechanisms and suggests that some degree of mitochondrial stressor as represented by mtROS may be critical to appropriately engage the mitochondrial quality control pathways. On the other hand, other studies have demonstrated that the mtROS produced downstream of iNOS in murine endotoxemia models are associated with increased markers of liver damage, suggesting that exuberant induction of ROS-generating pathways may be detrimental [22]. Similar data suggest that induction of SOD2 following LPS exposure, while ostensibly part of an antioxidant response, actually can contribute to an abundance of free radical damage through overproduction of hydrogen peroxide and downstream ROS [23–25]. This underscores the “double-edged sword” of free radical production as a signaling cascade: too little, and there is not a sufficient mitochondrial signal for healing (as demonstrated by our data); too much, and there is damage from the unfettered oxidative stress. Clinical data clearly suggests that patient responses during sepsis hinge upon the capacity of the mitochondrial networks in targeted organ systems to effectively remove damaged components, renew mitochondrial function, and regenerate healthy mitochondrial structure.

Our study provides further insight into the adaptive response of hepatocytes in response to LPS with a specific emphasis on mitochondrial regulation. We have demonstrated this using multiple approaches, including genetic manipulation and biochemical strategies, to outline some of the critical steps leading to the initiation of mitochondrial quality control following LPS exposure. While clinically, sepsis represents a broader range of insults and cellular dysfunction than the pure endotoxemia model utilized here, these data still provide important foundational insights and provide a framework for further exploration using both animal models of sepsis and human clinical sample data. Moreover, modulation of these pathways may offer therapeutic avenues to optimize cellular energetics, minimize cellular dysfunction, and potentially improve outcomes in sepsis.

## Data Availability

The appropriate data used to support the findings of this study are included within the article.
